# Shoulder Proprioception: A Review

**DOI:** 10.3390/jcm13072077

**Published:** 2024-04-03

**Authors:** Jake A. Fox, Lauren Luther, Eden Epner, Lance LeClere

**Affiliations:** Department of Orthopaedic Surgery, Vanderbilt University Medical Center, 1215 21st Ave S Ste 3200, Nashville, TN 37232, USA; jacob.a.fox.1@vumc.org (J.A.F.); lauren.luther@vumc.org (L.L.); l.leclere@vumc.org (L.L.)

**Keywords:** shoulder, proprioception, goniometer, inclinometer, motion analysis, rotator cuff, biceps tendon, return to play, glenohumeral, instability

## Abstract

The purpose of this review is to provide a comprehensive resource for shoulder proprioception assessment and its integration into clinical decision making as well as targeted rehabilitation protocols. Data for this review were acquired from peer-reviewed articles from computerized online databases, namely PubMed and Medline, published between 1906 and 2021. The development of digital/smart phone goniometers can improve shoulder joint range of motion (ROM) measurements and demonstrate comparable measurement accuracy to the universal standard goniometer. The inclinometer offers a portable and cost-effective method for measuring shoulder joint angles and arcs of motion in the vertical plane. Two types of dynamometers, the computerized isokinetic machine and the handheld hydraulic dynamometer, are reliable tools for objective shoulder rotator cuff strength assessment. Motion analysis systems are highly advanced modalities that create three-dimensional models of motion arcs using a series of cameras and reflective beads, offering unparalleled precision in shoulder proprioception measurement; however, they require time-consuming calibration and skilled operators. Advancements in wearable devices and compact mobile technology such as iPhone applications may make three-dimensional motion analysis more affordable and practical for outpatient settings in the future. The complex interplay between proprioception and shoulder dysfunction is not fully understood; however, shoulder proprioception can likely both contribute to and be caused by shoulder pathology. In patients with rotator cuff tears, glenohumeral osteoarthritis, and shoulder instability, clinicians can track proprioception to understand a patient’s disease progression or response to treatment. Finally, rehabilitation programs targeting shoulder proprioception have shown promising initial results in restoring function and returning athletes to play.

## 1. Introduction

This study aims to provide a comprehensive resource for assessing shoulder proprioception and integration into clinical decision making. We detail existing shoulder proprioception measurement modalities, their advantages/disadvantages, and discuss the bidirectional relationships between proprioception and common shoulder pathologies. Finally, we highlight the clinical importance of assessing shoulder proprioception for targeted rehabilitation protocols.

Proprioception is a concept first described by Charles Sherrington in 1906 as the human body’s “perception of joint movement and positioning in space in the absence of visual feedback” [1]. Since then, our understanding of proprioception has improved, defining it now as afferent information arising from peripheral areas of the body that contributes to joint stability, postural control, and motor control. Proprioception now comprises three subgroups—kinesthesia, joint position sense, and sensation of force [2,3]. Kinesthesia is the ability to consciously appreciate and interpret joint motion, while joint position sense refers to the body’s understanding of joint positions in space. The sensation of force allows us to appreciate force generated within a joint [4].

Proprioception relies on mechanoreceptors acting as transducers that convert mechanical energy into electrical nerve impulses. The central nervous system analyzes the impulse’s rate and frequency to interpret joint motion and position [5]. Several studies have found Ruffini and Pacinian-like corpuscles, free nerve endings, and Golgi mechanoreceptors within the shoulder joint’s capsuloligamentous structures [5,6,7,8]. Mechanoreceptors and proprioception play critical roles in healthy shoulder function. For instance, shoulder instability is associated with diminished proprioception, which can normalize after surgical reconstruction [9]. Shoulder stability involves both passive and dynamic components. Passive structures include the bony architecture, relative intra-articular pressure, capsuloligamentous structures, and the glenohumeral labrum. The dynamic aspects include muscular contraction coordinated around the joint and modulation from the neuromuscular system [10]. Effective proprioception from interactions between these components improves motor control and task performance [11].

Conscious proprioception is essential for fine motor skills and volitional activities, while unconscious proprioception influences reflex responses and joint stability [12]. Proprioceptive dysfunction correlates with higher rates of musculoskeletal injury, recurrence, and persistence of disability [9,13,14]. Proprioception is especially critical in the shoulder due to its unique anatomy, which sacrifices stability for increased ROM due to the size mismatch between the humeral head and glenoid [15].

Since the shoulder is the most mobile joint, and therefore most prone to instability, various physiotherapy (PT) protocols have been developed to combat this over the years. When implemented correctly, PT has been shown to improve shoulder joint position sense and proprioception. Typically, physiotherapy is incorporated as a conservative treatment effort for shoulder instability and impingement prior to surgical intervention. The goal of PT overall is to restore shoulder joint mobility, control, joint position sense, strength, and functioning. Specifically, physiotherapists can work with patients and athletes to restore proper scapular positioning by coordinating, strengthening, and stabilizing periscapular and shoulder musculature in order to maximize glenohumeral contact and stability. One study by Stokes et al. showed that rehabilitation efforts improve the patient’s scapular positioning, strength, and overall function [16]. These findings resembled another study by Salles et al., which demonstrated that an eight-week strength training program consisting of four shoulder exercises improved athletes’ neuromuscular control and shoulder joint position sense [17]. Another study by Jung et al. even demonstrated that a series of active shoulder exercises reduced shoulder subluxation and joint proprioception immediately following a stroke episode [18]. Thus, there are various non-surgical methods to improve shoulder laxity and instability in the appropriate patient population.

Although alterations in knee and ankle proprioception caused by musculoskeletal disorders have been extensively studied, existing research on shoulder proprioception focuses on specific pathologies [19,20]. For example, existing studies have examined proprioception in conjunction with rotator cuff tears (RCTs), frozen shoulder, glenohumeral joint instability, subacromial impingement, and glenohumeral arthritis [21,22,23,24,25,26,27,28,29,30,31]. This article aims to provide a more comprehensive resource on shoulder proprioception assessment, interpretation, and treatment for orthopedic, exercise, and sports health professionals.

## 2. Materials and Methods

Data for this review were acquired from peer-reviewed articles from computerized online databases, namely PubMed and Medline, published between the years 1906 and 2021. The key search terms utilized include “shoulder” AND “proprioception” as well as “return to play” AND “rotator cuff” and “glenohumeral joint” AND “instability.” Data were then evaluated and synthesized for the purposes of this review.

## 3. Results

### 3.1. Technology Utilized for Proprioception Measurement

#### 3.1.1. Goniometer

The universal goniometer is a simple device used for decades to measure joint ROM and considered the gold standard [23]. It consists of two arms: the stationary and the mobile arm. The stationary arm is placed over a select body part using anatomical landmarks, and the movable arm is rotated until it aligns with a second set of landmarks. The angle between these two arms indicates the joint motion achieved. This device is commonly used due to its low cost and accessibility [32]. However, its disadvantage is that it requires both examiners for proper measurements during examination. It is difficult to stabilize the arm during measurement, leading to increased human error and inaccuracy [23]. Measurement errors may also arise from inappropriate placement of the goniometer axis of rotation or inaccurate localization of bony landmarks [33].

Due to the rudimentary goniometer’s flaws, various modified forms of goniometry that attempt to improve on the original model have been described. Pérez-de la Cruz et al. evaluated a new, low-cost form of digital goniometer coined the “Hawk” goniometer, which measures joint ROM, velocity, and acceleration with resolution to one degree, similar to the universal goniometer [34]. The study found similar interobserver reliability and agreement estimates between the two devices, supporting the validity, reliability, and precision of the electronic goniometer [35,36,37].

Technological advances have led to the development of mobile applications that serve as goniometers. Johnson et al. conducted a comparative study, comparing the standard universal goniometer to a smartphone magnetometer-based goniometer application using the device’s built-in three-axis magnetometer. Both instruments demonstrated comparable measurement errors. The inter-rater concordance correlation coefficients (CCC) for the smartphone magnetometer’s ROM measurements were highly correlated with the predetermined ROM values in seated (CCC = 0.995) and supine positions (CCC = 0.989) [38]. This study, along with other studies investigating device-based applications for proprioception measurement, shows promising developments to enhance ROM measurement feasibility in the clinic setting [33,39].

#### 3.1.2. Inclinometer

**Figure 1:**
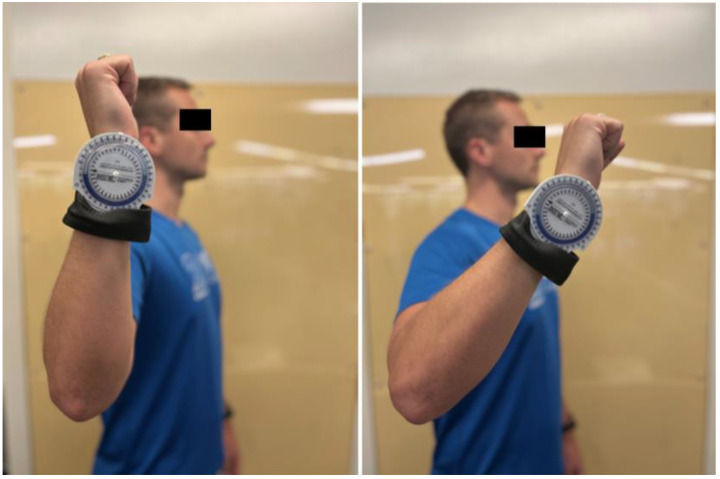
Inclinometer, utilized to measure shoulder arc of motion in vertical plane.

An inclinometer is an instrument used for measuring angles of slope, elevation, or depression using gravity as a reference point to assess joint motion (Figure 1) [40,41]. Its portable design and affordable cost make it widely used for assessing shoulder motion in clinics and training rooms [42,43]. Existing research found inclinometers produce reliable measurements in multiple arcs of motion [38,44,45]. One study observed low intra-rater variability in inclinometer measurements and sensitivity to detect small changes in external rotation (five degrees), internal rotation (four degrees), and posterior shoulder tightness (eight degrees) [46]. A 2010 meta-analysis established the inclinometer’s clinical applicability when it used inclinometers to assess physiologic movement of the shoulder in order to make more reliable decisions concerning shoulder joint restrictions [47].

Compared to goniometers, inclinometers offer several advantages. Inclinometers require only one hand for use, allowing for easier stabilization of the extremity being tested [48]. Inclinometers also demonstrate more accuracy and precision than goniometers in measuring ROM. Konnor et al. found inclinometer measurements of ankle ROM resulted in higher reliability coefficients (0.96–0.99) than goniometer measurements (0.85–0.96) [49]. Similarly, Hancock et al. determined inclinometers detect a minimal change of six degrees compared to ten degrees for goniometers [50]. Therefore, inclinometer and goniometer measurements should not be used interchangeably when evaluating a patient [48,51].

One significant disadvantage of inclinometers is their restriction to measuring ROM in the vertical plane due to their reliance on gravity [48]. They must be set to an accurate zero point before use to avoid improper referencing and incorrect measurements. Additionally, inclinometers provide two-dimensional data points, which may not capture dynamic shoulder movements as well as more sophisticated techniques. Despite the inclinometer’s high intra-observer reliability, several studies have demonstrated lower inter-observer reliability, complicating interpretation of results performed by different personnel [52].

#### 3.1.3. Isokinetic Dynamometer

Dynamometers assess force and power production from isolated muscle groups. This metric is crucial for evaluating muscle strength imbalance, which affects overall proprioception and ability to sense force generated through a joint. Numerous studies use dynamometers to assess shoulder and upper extremity proprioception and strength [22,42,53,54,55,56,57,58,59].

**Figure 2:**
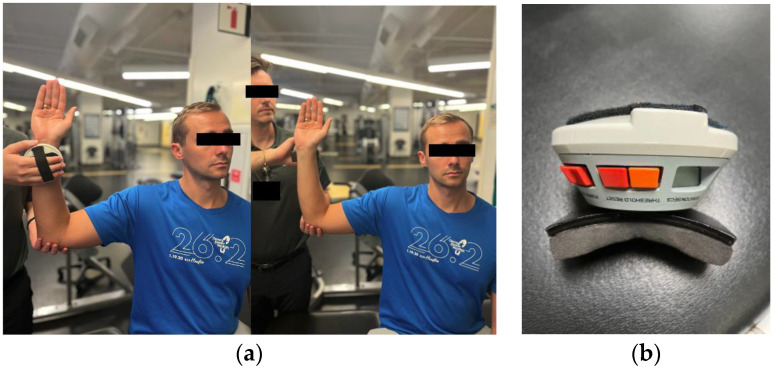
Isokinetic Dynamometer: (**a**) demonstration of measuring power production from isolated muscle groups utilizing handheld dynamometer; (**b**) handheld dynamometer device.

Two types of dynamometers exist, one of which is a fixed, computerized, and cumbersome isokinetic machine (eg. Cybex, Biodex, and KinCom) that measures several muscle force output parameters [56]. The second type is a handheld hydraulic dynamometer (eg. MicroFET2, MicroFET3, Nicholas manual muscle tester) that offers limited peak force data, portability, and ease of use with minimal training (Figure 2) [53,56].

Isokinetic machines are historically considered the gold standard for measuring peak force, endurance, power, and angle of maximal force precisely and with data-driven strength curves [56,60]. However, they have significant cost, time-consuming installation, and intricate protocols, rendering them unrealistic for most health professionals [60].

The convenience of handheld dynamometers comes at the cost of an output dataset limited to peak force, time to peak force, and total test duration. They cannot produce strength curve profiles, estimate power like their computerized counterparts, nor provide positional information on the joint or extremity being tested [56]. However, studies by Bohannon et al. and Stratford & Balsor examining make-and-break tests show handheld dynamometry is a viable alternative to costly isometric strength machines (Cybex and Kin-Com) if the examiner’s strength exceeds the muscle being measured [58,61]. Stratford & Balsor also found no significant difference in the reliability coefficients obtained from the Kin-Com test compared to the handheld dynamometer (*p* > 0.05) [58]. A 2011 systematic review further reinforces handheld dynamometer testing’s validity for assessing upper extremity strength [62]. Another systematic review agrees that handheld dynamometers are reliable tools for shoulder rotator cuff strength assessment but urges caution due to clinical heterogeneity and method flaws [63].

Historically, handheld dynamometers lacked sufficient normative data for comparison. To address this, Cools et al. published a paper describing a reference database of eccentric rotator cuff strength for athletes [64]. Further research is needed to establish robust reference values for strength for other shoulder movements and patient populations. Despite their limitations compared to sophisticated isokinetic machines, handheld dynamometers remain a viable option for objective strength assessments, particularly in smaller community settings.

#### 3.1.4. Motion Analysis

**Figure 3:**
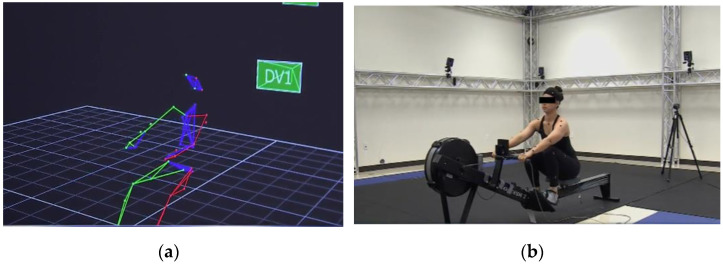
Motion analysis: (**a**) 3D computerized model of joint motion; (**b**) model with reflective motion analysis beads attached to joints of interest.

Motion analysis systems are the most technologically advanced tools for measuring extremity ROM. They create three-dimensional models of motion arcs, unlike goniometers and inclinometers, which are two-dimensional. Barriers to accessing this technology include significant equipment cost, installation and maintenance time, and the expertise required for appropriate functioning. As a result, these systems are less frequently used to measure proprioception and have limited data in the literature (Figure 3) [25,27,28,45,65,66,67].

Additionally, motion analysis provides an unparalleled level of precision in proprioception measurement. For instance, the Vicon motion capture system consists of 12 infrared video cameras with a resolution of 1.3 megapixels and a recording frequency of 120 Hz with a range of +/− one millimeter (Figure 3) [25]. These cameras capture and synthesize reflected infrared light from eight-millimeter markers placed on each patient’s body. Reflections must be recorded by at least two cameras to create a three-dimensional model from two-dimensional information. Before each analysis, the system needs recalibration both with static and dynamic calibration, requiring time and a skilled operator.

**Figure 4:**
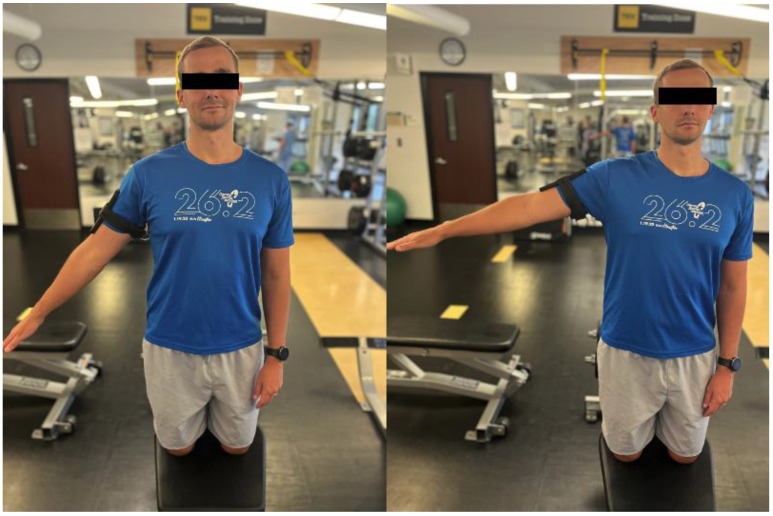
Compact wearable mobile device (iPhone/iPod touch) recording three-dimensional shoulder joint motion.

Myers and Lephart also used a six degrees-of-freedom electromagnetic motion analysis system to assess athletes’ ability to replicate a motor pathway by measuring the three-dimensional variations between the presented and reproduced paths of motion [4]. Although this electromagnetic motion analysis modality is useful, larger static modalities like these are increasingly being replaced by more compact mobile technology and wearable devices. One such novel study used more accessible technology—an iPod touch (Figure 4) [68]. Leveraging internal sensors like gyroscopes and accelerometers, they recorded the phone’s orientation with respect to gravity while strapped to an extremity. With rapid innovation in the wearable device realm, similar three-dimensional motion analysis may become more affordable and realistic options for outpatient settings.

## 4. Discussion

### 4.1. Clinical Applications: Rotator Cuff

The rotator cuff is a critical structure for shoulder proprioception and overall glenohumeral joint function. Johansson et al. conducted eccentric testing of rotator cuff strength using a handheld dynamometer. They found excellent intratester reliability (ICC = 0.87–0.85) and good intertester reliability (ICC = 0.71) [53]. The concurrent validity of their handheld dynamometer protocol with the gold standard (Biodex) was good to excellent, varying from 0.7–0.78, aligning with previous studies on isometric shoulder strength using a hand dynamometer [69]. Properly evaluating cuff strength and proprioception is crucial for overhead athletes, as preseason external rotation weakness correlates with supraspinatus deficits and future injury [70]. Gumina et al. conducted a case-control study evaluating rotator cuff tears’ (RCTs) effects on shoulder proprioception. Patients in their study were instructed to actively move their shoulder into various degrees of flexion (30°, 60°, 90°, 120°, 150°) measured by an inclinometer, then actively reproduce the same angles while blindfolded. Patients with RCTs were significantly worse than controls at finding the same joint position at all angles measured (*p* < 0.05) [24]. As the size of the RCT increased, the measured absolute error rose at a significantly increased rate, possibly due to a high concentration of muscle spindles and golgi tendon organs in the cuff complex [71,72]. Additional studies bolster the concept that RCT is correlated with a decline in shoulder proprioception. Safran et al. demonstrated impaired kinesthesia in overhead throwers with cuff tendinopathy and suggested increased pain from nociceptors in the painful shoulder may override the accuracy of proprioceptive input. Patients with painful RCT also have impaired sensation of force through their shoulder joint, causing them to overestimate targeted forces and produce higher forces than necessary in external and internal rotation [73]. The medical literature lacks studies on the effect of rotator cuff repair on postoperative shoulder proprioception. Comparative studies of shoulder proprioceptive ability before and after repair could shed light on whether operative fixation resolves the proprioceptive deficit and its potential impact on re-tear following repair.

### 4.2. Clinical Applications: Glenohumeral Arthritis

Proprioception has also been studied in patients undergoing shoulder arthroplasty. Cuomo et al. conducted a prospective analysis on 20 patients with unilateral advanced glenohumeral arthritis who underwent total shoulder arthroplasty. In their study, they evaluated passive motion sense and ability to detect motion one week before surgery and six months after joint replacement. They found that osteoarthritis had a significant effect on position sense in flexion, abduction, and external rotation. Their unaffected shoulder could reproduce set angles of the three aforementioned movements within 5.2°, 5.4°, and 5.8°, respectively. The arthritic shoulder produced the same accuracy, averaging 7.1°, 7.3°, and 8.2° in the three planes, respectively (*p* < 0.05). Following surgery, the replaced shoulder showed significant improvement in joint position sense compared to preoperative baseline (*p* < 0.05). Also, the postoperative shoulders also had no significant difference in proprioceptive function from the uninvolved contralateral side at six months. The authors postulated that diminished afferent input from pain fibers may allow increased sensitivity and recognition of proprioceptive signals. They also hypothesized that extracapsular factors may play a larger role in position sense than previously thought, such as the restoration of capsule and muscle tension [21]. This study aligns with a study on knee proprioception in patients with knee osteoarthritis, which demonstrated diminished joint position sense before knee replacement surgery and subsequent increased accuracy in angle reproducibility postoperatively [74]. Improved joint position sense following shoulder replacement has also been observed in patients following reverse total shoulder arthroplasty. Walecka et al. conducted a retrospective study on 29 patients with unilateral shoulder osteoarthritis who underwent reverse total shoulder arthroplasty compared to a control group. They found that patients who underwent shoulder replacement showed similar active joint position matching in the operated shoulder compared to the healthy control group. Additionally, they found superior joint position matching in the operative shoulder in flexion (30°, 90°, and 120°), abduction (30°, 90°, and 120°), internal rotation (30°, 45°), and external rotation of 15° compared to the contralateral uninvolved shoulder. All patients in their cohort were satisfied with the functional outcome from their procedure [31]. However, one study examining 26 patients who underwent total shoulder arthroplasty (n = 13), hemi-arthroplasty (n = 8), or reverse arthroplasty (n = 5) did not find improvement in shoulder proprioception post-operatively [27]. Examiners studied patients before the operation and then again six months after using motion analysis testing on active angle reproduction. Active reproduction worsened at 60° of flexion from 5.5° to 7.6° (*p* = 0.007) and at 30° of external rotation from 6.5° to 7.3° (*p* = 0.023) six months following surgery. They compared their findings to the prior study by Cuomo et al. and theorized that the lack of pain afferent information these patients are accustomed to may counterintuitively adversely influence their performance with active reproduction after surgery. Maier et al. evaluated proprioception in the shoulder three years following total or hemiarthroplasty and similarly found a 3.7° significant deterioration of active angle reproduction following shoulder replacement surgery. They also postulated that postoperative lack of pain leads to difficulty with shoulder proprioception, as these patients are accustomed to relying on pain [75]. Another possible explanation is that surgical approaches may alter the proprioceptive ability of the shoulder depending on the anatomic structures that are disrupted. The mixed findings in the literature regarding shoulder arthroplasty and proprioception require further studies to elucidate their relationship. Preliminary research indicates a lack of proprioception prevents shoulder arthroplasty patients from utilizing the full potential of their range of motion restored post-operatively [27]. Additionally, with the expanding indications for reverse total shoulder arthroplasty, pre-operative proprioceptive testing could help triage patients to different arthroplasty options [76].

### 4.3. Clinical Applications: Shoulder Instability

One active area of research on shoulder proprioception is in patients with shoulder instability. Hung and Darling compared ten patients with anterior shoulder instability to 15 healthy controls in active angle reproduction and passive matching [26]. They found that individuals with instability had significantly larger errors (1.8° on average) in perceiving shoulder position compared to controls in passive matching of set angles. However, the injured patients showed no difference from controls in active angle reproduction. Potzl et al. [28] performed a prospective study on 14 patients with recurrent anterior shoulder instability, evaluating their active angle reproduction preoperatively and five years postoperatively. They found that patients with instability preoperatively had significantly decreased ability to actively meet a target joint position in flexion, abduction, and external rotation. However, at the five-year follow-up after shoulder reconstruction, joint position sense significantly improved in all three planes of movement (*p* < 0.05). On average across all movement planes, patients got within 4.1° of the set target angle. The authors argue that the restored shoulder stability leads to improved shoulder proprioception [28]. Despite the improved proprioception noted postoperatively, three out of 14 patients experienced a recurrent dislocation event in the postoperative period. This reported dislocation rate is at the higher end of a previous systematic review that reported recurrent instability rates of 11–24% following Bankart repair [77]. Potzl et al. examined a small cohort of patients, but this still highlights the fact that the improved proprioception may not be a protective factor for recurrent dislocation. Interestingly, the study conducted by Potzl and colleagues found the contralateral shoulder of operative patients also had significant improvement in position sense of their previously healthy shoulder. This finding highlights that joint position sense is not only regulated peripherally but also centrally, meaning improvement on one side of the body can positively influence the contralateral side [28]. Lubiatowski et al. reported a similar phenomenon in patients with unilateral shoulder instability, demonstrating deficits of active reproduction of joint position in both the injured and healthy, uninvolved shoulders [78]. Machner and colleagues had similar results in patients with anterior shoulder instability who underwent arthroscopic labrum repair. Preoperatively, patients had diminished proprioception, but at 18 months follow up, the repaired shoulders showed improvement to the point that movement sense was not different from uninjured shoulders [79]. Aydin et al. also studied 20 patients with instability who underwent surgical repair and found no significant difference in joint position sense between the surgical shoulder and the contralateral healthy shoulder, further reinforcing the benefits of surgical repair for proprioception [12].

### 4.4. Clinical Applications: Biceps Tendon

The tendon of biceps brachii is one of multiple tendons in the body to cross two joints. At the elbow joint, it functions primarily as a supinator and secondarily as an elbow flexor [80]. At the shoulder, the biceps functions as a dynamic stabilizer and depressor of the humeral head and an elevator of the glenoid labrum [9,81]. However, the true function and proprioceptive properties of the biceps are less known and have been evaluated with various electromyographic (EMG) studies [82,83,84]. In EMG analysis conducted on ten patients by Levy and colleagues, they found no activity in the long head of the biceps (LHB) tendon during shoulder movement when the elbow and forearm were locked in a static position [84]. This study was different from many previous studies as they removed the variables of elbow/forearm motion. They concluded that the function of the LHB tendon is either achieved passively through shoulder proprioception or actively in association with elbow and forearm motion. Glousman et al. studied the EMG properties of the biceps brachii in 15 overhead throwing athletes. They reported mildly increased EMG activity in the biceps tendon in those with anterior shoulder instability compared to uninjured controls [82]. The team then concluded that the biceps tendon may enhance shoulder proprioception by stabilizing the humeral head against the glenoid. Ghalayini et al. studied three patients with congenital absence of the LHB tendon and believe, based on their study and previous EMG studies, that the biceps does have important proprioceptive properties in stabilizing the humeral head [85]. Currently, there are no published studies evaluating proprioception in patients that have undergone biceps tenodesis or tenotomy. There is much unknown about the biceps and its role in shoulder stability and proprioception, but the studies above highlight the potential importance of the long head of biceps in overall shoulder proprioception.

### 4.5. Clinical Applications: Rehabilitation Protocols

Rehabilitation Protocols Rehabilitation for upper extremity injuries should initially address inflammation and pain, restore of ROM and flexibility, and strength through traditional exercises. However, given its importance in shoulder function, clinicians and therapists can also employ directed rehabilitation protocols aimed at improving proprioception. Proprioceptive exercises create a tensile loading mechanism, such as weight-bearing wobble board and flexible foil training, stimulating the articular and muscular receptors (Meissner’s corpuscle, Pacinian corpuscle, ruffini endings, muscle spindles) directly [86]. A potential secondary benefit is an indirect increase in mechanoreceptor inputs of nearby structures, such as the joint capsule, cutaneous tissues, and ligaments. Several rehabilitation programs targeting shoulder proprioception follow a three-phase, progressive framework. The first phase prescribes static stabilization exercises: balance with both hands on the floor in isometric contraction and balance with one hand clockwise on the wall. Phase two incorporates more advanced stabilization exercises: double arm balance in kneeling push-up position on a balance board, rotation of the hand on the wall using a ball, and double arm balance in kneeling push-up position on foam. Phase three combines static and dynamic stabilization with one hand: scapular stabilization on the floor with one hand in isometric contraction and dynamic stabilization exercise on a ball with one hand. By following this protocol, subjects saw a significant improvement in sense of kinesthesia at ten degrees external rotation, as well as reproduction of active and passive positioning at ten degrees of external rotation [87]. Atya et al. found that passive therapies, such as six weeks of micro-cutaneous electrical nerve stimulation, did not significantly improve passive joint position sense (*p* = 0.67) [88]. Bracing also showed no improvement in active joint position sense in 90 degrees of abduction or 30 degrees of internal rotation/external rotation (*p* > 0.05) according to a study by Chu et al. [89]. Similarly, kinesiology taping does not seem to significantly improve shoulder proprioception [68,90,91]. Passive methods may be less effective due to weaker stimulation of mechanoreceptors. Functional rehabilitation is essential when preparing athletes for return to play. First developed by Lephart and Henry, this framework focuses on restoring proprioceptive function and neuromuscular control of the glenohumeral joint prior to resuming high-performance activities [54]. Rehabilitation activities mimic the demands placed on the shoulder during sport, increasing sensitivity of peripheral afferents in capsuloligamentous structures, reestablishing afferent pathways, facilitating coactivation of the force couples, eliciting preparatory and reactive muscle contractions, and increasing muscle stiffness [41]. Lephart and Myers provided a strong foundation for clinicians to follow in this type of rehabilitation program [4]. The program addresses awareness of proprioception, dynamic-stabilization restoration, preparatory and reactive muscle facilitation, and replication of functional activities. However, the efficacy of their exercises is limited to anecdotal evidence, and controlled trials are needed to explore the validity of their model further.

### 4.6. Future Directions

While commonly employed in evaluation of lower extremity proprioception, force plate analysis is a relatively new tool for upper extremity proprioception analysis. Force plates are measurement platforms that utilize piezoelectric force sensors to measure ground reaction forces. One primary test with these plates is sway velocity, where a patient holds a one-arm plank position on the force plate for 20 s and then switches to the contralateral upper extremity. Pontillo and Sennett have been prominent early investigators of the force plates, defining sway velocity as the speed at which the center of pressure moves, indicating the patient’s ability to stabilize their arm during the task [92]. They assess the sway in both the antero-posterior and medial-lateral planes, which is important because it evaluates several facets of arm function simultaneously: ability to withstand shear/compressive forces through all joints, co-contraction of upper extremity muscles, and core stability [92]. In their study, they used a force plate system (Kistler Inc.; Amherst, NY, USA) with connected analysis software (Sparta Science, Menlo Park, CA, USA) and found moderate to excellent reliability of the force plates to assess sway velocity. Their analysis of healthy athletes determined no difference between extremities in any studied variables, potentially indicating asymmetry as a predictor of injury. The long-term goal of the force plates is to first determine how sway analysis correlates with upper extremity injury and then potentially use the plates in return to play decision making. Another technology that has shown promise in upper extremity proprioceptive rehabilitation is the wearable arm, or upper extremity, exoskeleton. These devices have commonly been evaluated in stroke patients but could potentially be helpful for athletes with proprioceptive deficits from injury. The most widely used wearable arm has a purely mechanical structure involving joints driven by motors, which helps offset the load of gravity [93]. These devices measure upper limb movement, assist in upper extremity rehabilitation, and can be useful for specific high-precision tasks [94,95]. The wearable arm can provide real time vibrotactile feedback, measuring the movement of your own arm, as well as reliable trajectory measurement. One disadvantage of these devices is the high cost and complexity of associated algorithms [95,96]. The wearable arm has not made its way into the athletic population for proprioception-related studies, but its capabilities could be promising in the future.

## 5. Conclusions

Proprioception has been discussed in the medical literature since 1906, but our understanding of its clinical application and utility continue to evolve. Various options exist for measuring proprioception, each with relative advantages and disadvantages. The complex interplay between proprioception and shoulder dysfunction is not fully understood, but it is likely that impaired shoulder proprioception can both contribute to and be caused by shoulder pathology. In patients with rotator cuff tears, glenohumeral osteoarthritis, and shoulder instability, clinicians can track proprioception to understand a patient’s disease progression or response to treatment. Finally, rehabilitation programs targeting shoulder proprioception have shown promising initial results in restoring function and returning athletes to play.

### Current Literature Gaps

The medical literature has made significant progress in defining, evaluating, and testing proprioception in the last two decades. However, some gaps still exist. Firstly, the correlation between proprioception and subsequent shoulder injury remains largely unknown. Secondly, there are currently no studies detailing patient-reported outcomes following postoperative improvement of shoulder proprioception. It is crucial to understand the patient’s perception of their improved shoulder proprioception postoperatively. Thirdly, current literature lacks robust prospective or randomized controlled trials conducted on specific shoulder proprioception rehabilitation protocols. Finally, a relatively uncharted territory involves wearable upper extremity devices to help patients with poor proprioception function at a higher level.

## Data Availability

No new data was created in this study.
